# Supplementary material for: Disentangling the Genetic Landscape of Peripartum Depression: A Multi-Polygenic Machine Learning Approach on an Italian Sample
Source: Genes (Basel). 2024 Nov 26;15(12):1517. doi: 10.3390/genes15121517 (PMC11675425; doi:10.3390/genes15121517)
Supplement: Supplementary file 1 [file genes-15-01517-s001.zip › genes-3318496-supplementary.pdf]

SUPPLEMENTARY MATERIALS AND METHODS

Supplementary Table S1. Full list of polygenic risk scores (PRSs) included in all partial least squares regression analyses.

**PRS category**	**PGS Catalog code and trait**	**Link to PGS Catalog score**
Psychiatric symptoms and disorders	PGS000133 Schizophrenia	https://www.pgscatalog.org/score/PGS000133/
	PGS000135 Schizophrenia	https://www.pgscatalog.org/score/PGS000135/
	PGS000134 Schizophrenia	https://www.pgscatalog.org/score/PGS000134/
	PGS000136 Schizophrenia	https://www.pgscatalog.org/score/PGS000136/
	PGS002785 Schizophrenia	https://www.pgscatalog.org/score/PGS002785/
	PGS000138 Lifetime Major Depressive Disorder	https://www.pgscatalog.org/score/PGS000138/
	PGS000139 Lifetime Major Depressive Disorder (with recurrence)	https://www.pgscatalog.org/score/PGS000139/
	PGS000140 Broad Depression (seen a General Practitioner for nerves, anxiety, tension or depression)	https://www.pgscatalog.org/score/PGS000140/
	PGS000141 Seen a psychiatrist for nerves, anxiety, tension or depression	https://www.pgscatalog.org/score/PGS000141/
	PGS000142 Probable Depression (low mood or anhedonia, and seen a GP or psychiatrist for nerves, anxiety, tension or depression)	https://www.pgscatalog.org/score/PGS000142/
	PGS000143 Seen a General Practitioner for nerves, anxiety, tension or depression (without report of low mood or anhedonia)	https://www.pgscatalog.org/score/PGS000143/
	PGS000144 Self-reported depression or depression symptoms	https://www.pgscatalog.org/score/PGS000144/
	PGS000145 Depression (ICD-10 defined)	https://www.pgscatalog.org/score/PGS000145/
	PGS000193 Major depressive disorder	https://www.pgscatalog.org/score/PGS000193/
	PGS000907 Major depressive disorder	https://www.pgscatalog.org/score/PGS000907/
	PGS000767 Depression	https://www.pgscatalog.org/score/PGS000767/
	PGS001829 Depression	https://www.pgscatalog.org/score/PGS001829/
	PGS002759 Depression	https://www.pgscatalog.org/score/PGS002759/
	PGS003576 Major depressive disorder (lifetime)	https://www.pgscatalog.org/score/PGS003576/
	PGS003577 Major depressive disorder (lifetime)	https://www.pgscatalog.org/score/PGS003577/
	PGS003578 Major depressive disorder (lifetime)	https://www.pgscatalog.org/score/PGS003578/
	PGS003579 Major depressive disorder (lifetime)	https://www.pgscatalog.org/score/PGS003579/
	PGS003580 Major depressive disorder (lifetime)	https://www.pgscatalog.org/score/PGS003580/
	PGS003581 Major depressive disorder (lifetime)	https://www.pgscatalog.org/score/PGS003581/
	PGS003582 Major depressive disorder (lifetime)	https://www.pgscatalog.org/score/PGS003582/
	PGS003583 Major depressive disorder (lifetime)	https://www.pgscatalog.org/score/PGS003583/
	PGS003584 Major depressive disorder (lifetime)	https://www.pgscatalog.org/score/PGS003584/
	PGS003585 Major depressive disorder (lifetime)	https://www.pgscatalog.org/score/PGS003585/
	PGS002036 Depression	https://www.pgscatalog.org/score/PGS002036/
	PGS002789 Major depressive disorder	https://www.pgscatalog.org/score/PGS002789/
	PGS003333 Major depressive disorder	https://www.pgscatalog.org/score/PGS003333/
	PGS003442 Major depressive disorder	https://www.pgscatalog.org/score/PGS003442/
	PGS004591 Major depressive disorder	https://www.pgscatalog.org/score/PGS004591/
	PGS002786 Bipolar disorder	https://www.pgscatalog.org/score/PGS002786/
	PGS002787 Type 1 bipolar disorder	https://www.pgscatalog.org/score/PGS002787/
	PGS002788 Type 2 bipolar disorder	https://www.pgscatalog.org/score/PGS002788/
	PGS002746 ADHD	https://www.pgscatalog.org/score/PGS002746/

	PGS003753 ADHD	https://www.pgscatalog.org/score/PGS003753/
	PGS000327 Autism spectrum disorder	https://www.pgscatalog.org/score/PGS000327/
	PGS002790 Autism spectrum disorder	https://www.pgscatalog.org/score/PGS002790/
	PGS001022 Worry too long after an embarrassing experience	https://www.pgscatalog.org/score/PGS001022/
	PGS001017 Feeling nervous measurement	https://www.pgscatalog.org/score/PGS001017/
	PGS001021 Feelings of worry or anxiety	https://www.pgscatalog.org/score/PGS001021/
	PGS001016 Sensitivity/hurt feelings	https://www.pgscatalog.org/score/PGS001016/
	PGS001091 Loneliness	https://www.pgscatalog.org/score/PGS001091/
	PGS001920 Recent feelings of foreboding	https://www.pgscatalog.org/score/PGS001920/
	PGS002136 Recent feelings of foreboding	https://www.pgscatalog.org/score/PGS002136/
	PGS000205 Risk taking tendency	https://www.pgscatalog.org/score/PGS000205/
	PGS000206 Risk taking tendency	https://www.pgscatalog.org/score/PGS000206/
	PGS001049 Risk taking behaviour	https://www.pgscatalog.org/score/PGS001049/
	PGS001996 Neuroticism	https://www.pgscatalog.org/score/PGS001996/
	PGS002213 Neuroticism	https://www.pgscatalog.org/score/PGS002213/
	PGS002342 Neuroticism	https://www.pgscatalog.org/score/PGS002342/
	PGS002414 Neuroticism	https://www.pgscatalog.org/score/PGS002414/
	PGS002463 Neuroticism	https://www.pgscatalog.org/score/PGS002463/
	PGS002512 Neuroticism	https://www.pgscatalog.org/score/PGS002512/
	PGS002561 Neuroticism	https://www.pgscatalog.org/score/PGS002561/
	PGS002610 Neuroticism	https://www.pgscatalog.org/score/PGS002610/
	PGS002659 Neuroticism	https://www.pgscatalog.org/score/PGS002659/
	PGS002708 Neuroticism	https://www.pgscatalog.org/score/PGS002708/
	PGS003565 Neuroticism	https://www.pgscatalog.org/score/PGS003565/
	PGS004430 Neuroticism	https://www.pgscatalog.org/score/PGS004430/
	PGS004608 Neuroticism	https://www.pgscatalog.org/score/PGS004608/
	PGS002113 Depression by trauma	https://www.pgscatalog.org/score/PGS002113/
	PGS001018 Social interaction	https://www.pgscatalog.org/score/PGS001018/
	PGS001019 Social interaction	https://www.pgscatalog.org/score/PGS001019/
	PGS001020 Social interaction	https://www.pgscatalog.org/score/PGS001020/
Hormones and pregnancy-related conditions	PGS001182 Estradiol	https://www.pgscatalog.org/score/PGS001182/
	PGS002914 Estradiol	https://www.pgscatalog.org/score/PGS002914/
	PGS002915 Estradiol	https://www.pgscatalog.org/score/PGS002915/
	PGS002916 Estradiol	https://www.pgscatalog.org/score/PGS002916/
	PGS002917 Estradiol	https://www.pgscatalog.org/score/PGS002917/
	PGS002918 Estradiol	https://www.pgscatalog.org/score/PGS002918/
	PGS002919 Estradiol	https://www.pgscatalog.org/score/PGS002919/
	PGS002920 Estradiol	https://www.pgscatalog.org/score/PGS002920/
	PGS002921 Estradiol	https://www.pgscatalog.org/score/PGS002921/
	PGS002922 Estradiol	https://www.pgscatalog.org/score/PGS002922/
	PGS002923 Estradiol	https://www.pgscatalog.org/score/PGS002923/
	PGS002820 Age at menopause	https://www.pgscatalog.org/score/PGS002820/
	PGS002821 Age at menopause	https://www.pgscatalog.org/score/PGS002821/
	PGS002822 Age at menopause	https://www.pgscatalog.org/score/PGS002822/

	PGS002823 Age at menopause	https://www.pgscatalog.org/score/PGS002823/
	PGS002824 Age at menopause	https://www.pgscatalog.org/score/PGS002824/
	PGS001912 Age first birth	https://www.pgscatalog.org/score/PGS001912/
	PGS002127 Age first birth	https://www.pgscatalog.org/score/PGS002127/
	PGS003505 Age first birth	https://www.pgscatalog.org/score/PGS003505/
	PGS002806 Gestational duration	https://www.pgscatalog.org/score/PGS002806/
	PGS002256 Gestational diabetes	https://www.pgscatalog.org/score/PGS002256/
	PGS003587 Gestational hypertension	https://www.pgscatalog.org/score/PGS003587/
	PGS003586 Preeclampsia	https://www.pgscatalog.org/score/PGS003587/
	PGS004593 Preeclampsia	https://www.pgscatalog.org/score/PGS004593/
	PGS001915 Age menarche	https://www.pgscatalog.org/score/PGS001915/
	PGS002131 Age menarche	https://www.pgscatalog.org/score/PGS002131/
	PGS002310 Age menarche	https://www.pgscatalog.org/score/PGS002310/
	PGS002382 Age menarche	https://www.pgscatalog.org/score/PGS002382/
	PGS002431 Age menarche	https://www.pgscatalog.org/score/PGS002431/
	PGS002480 Age menarche	https://www.pgscatalog.org/score/PGS002480/
	PGS002529 Age menarche	https://www.pgscatalog.org/score/PGS002529/
	PGS002578 Age menarche	https://www.pgscatalog.org/score/PGS002578/
	PGS002627 Age menarche	https://www.pgscatalog.org/score/PGS002627/
	PGS002676 Age menarche	https://www.pgscatalog.org/score/PGS002676/
	PGS002815 Age menarche	https://www.pgscatalog.org/score/PGS002815/
	PGS002816 Age menarche	https://www.pgscatalog.org/score/PGS002816/
	PGS002817 Age menarche	https://www.pgscatalog.org/score/PGS002817/
	PGS002818 Age menarche	https://www.pgscatalog.org/score/PGS002818/
	PGS002819 Age menarche	https://www.pgscatalog.org/score/PGS002819/
	PGS003508 Age menarche	https://www.pgscatalog.org/score/PGS003508/
	PGS002381 Number of kids	https://www.pgscatalog.org/score/PGS002381/
	PGS002430 Number of kids	https://www.pgscatalog.org/score/PGS002430/
	PGS002309 Number of kids	https://www.pgscatalog.org/score/PGS002309/
	PGS002479 Number of kids	https://www.pgscatalog.org/score/PGS002479/
	PGS002528 Number of kids	https://www.pgscatalog.org/score/PGS002528/
	PGS002577 Number of kids	https://www.pgscatalog.org/score/PGS002577/
	PGS002626 Number of kids	https://www.pgscatalog.org/score/PGS002626/
	PGS002675 Number of kids	https://www.pgscatalog.org/score/PGS002675/
	PGS002079 Irregular cycle	https://www.pgscatalog.org/score/PGS002079/
	PGS001866 Endometriosis	https://www.pgscatalog.org/score/PGS001866/
	PGS002077 Endometriosis	https://www.pgscatalog.org/score/PGS002077/
	PGS003447 Endometriosis	https://www.pgscatalog.org/score/PGS003447/
	PGS001883 Osteoporosis	https://www.pgscatalog.org/score/PGS001883/
	PGS001913 Length of menstrual cycle	https://www.pgscatalog.org/score/PGS001913/
	PGS002128 Length of menstrual cycle	https://www.pgscatalog.org/score/PGS002128/
	PGS002129 Length of menstrual cycle	https://www.pgscatalog.org/score/PGS002129/
	PGS003506 Length of menstrual cycle	https://www.pgscatalog.org/score/PGS003506/
	PGS001977 Sex hormone binding globulin	https://www.pgscatalog.org/score/PGS001977/

	PGS002195 Sex hormone binding globulin	https://www.pgscatalog.org/score/PGS002195/
	PGS003552 Sex hormone binding globulin	https://www.pgscatalog.org/score/PGS003552/
	PGS000694 Sex hormone binding globulin	https://www.pgscatalog.org/score/PGS000694/
	PGS004240 Sex hormone binding globulin	https://www.pgscatalog.org/score/PGS004240/
	PGS000217 Adrenomedullin	https://www.pgscatalog.org/score/PGS000217/
	PGS000240 Follistatin serum levels	https://www.pgscatalog.org/score/PGS000240/
	PGS000176 Placenta growth factor	https://www.pgscatalog.org/score/PGS000176/
	PGS000322 Testosterone levels in females	https://www.pgscatalog.org/score/PGS000322/
	PGS001914 Testosterone levels in females	https://www.pgscatalog.org/score/PGS001914/
	PGS002130 Testosterone in females	https://www.pgscatalog.org/score/PGS002130/
	PGS003507 Testosterone in females	https://www.pgscatalog.org/score/PGS003507/
	PGS000759 Hypothyroidism	https://www.pgscatalog.org/score/PGS000759/
	PGS000761 Hypothyroidism	https://www.pgscatalog.org/score/PGS000761/
	PGS000820 Hypothyroidism	https://www.pgscatalog.org/score/PGS000820/
	PGS001816 Hypothyroidism	https://www.pgscatalog.org/score/PGS001816/
	PGS002024 Hypothyroidism	https://www.pgscatalog.org/score/PGS002024/
	PGS002336 Hypothyroidism	https://www.pgscatalog.org/score/PGS002336/
	PGS002408 Hypothyroidism	https://www.pgscatalog.org/score/PGS002408/
	PGS002457 Hypothyroidism	https://www.pgscatalog.org/score/PGS002457/
	PGS002506 Hypothyroidism	https://www.pgscatalog.org/score/PGS002506/
	PGS002555 Hypothyroidism	https://www.pgscatalog.org/score/PGS002555/
	PGS002604 Hypothyroidism	https://www.pgscatalog.org/score/PGS002604/
	PGS002653 Hypothyroidism	https://www.pgscatalog.org/score/PGS002653/
	PGS002702 Hypothyroidism	https://www.pgscatalog.org/score/PGS002702/
	PGS002766 Hypothyroidism	https://www.pgscatalog.org/score/PGS002766/
	PGS000821 Thyroid med use	https://www.pgscatalog.org/score/PGS000821/
	PGS001298 Obesity (time to event)	https://www.pgscatalog.org/score/PGS001298/
	PGS001825 Obesity	https://www.pgscatalog.org/score/PGS001825/
	PGS002033 Obesity	https://www.pgscatalog.org/score/PGS002033/
	PGS003400 Obesity	https://www.pgscatalog.org/score/PGS003400/
	PGS003959 Obesity	https://www.pgscatalog.org/score/PGS003459/
Circadian rhythms and sleep-related problems	PGS000336 Chronotype measurement	https://www.pgscatalog.org/score/PGS000336/
	PGS001055 Chronotype	https://www.pgscatalog.org/score/PGS001055/
	PGS001992 Chronotype	https://www.pgscatalog.org/score/PGS001992/
	PGS002209 Chronotype	https://www.pgscatalog.org/score/PGS002209/
	PGS002318 Chronotype (morning)	https://www.pgscatalog.org/score/PGS002318/
	PGS002390 Chronotype (morning)	https://www.pgscatalog.org/score/PGS002390/
	PGS002439 Chronotype (morning)	https://www.pgscatalog.org/score/PGS002439/
	PGS002488 Chronotype (morning)	https://www.pgscatalog.org/score/PGS002488/
	PGS002537 Chronotype (morning)	https://www.pgscatalog.org/score/PGS002537/
	PGS002586 Chronotype (morning)	https://www.pgscatalog.org/score/PGS002586/
	PGS002635 Chronotype (morning)	https://www.pgscatalog.org/score/PGS002635/
	PGS002684 Chronotype (morning)	https://www.pgscatalog.org/score/PGS002684/
	PGS003563 Chronotype	https://www.pgscatalog.org/score/PGS003563/

	PGS000757 Narcolepsy	https://www.pgscatalog.org/score/PGS000757/
	PGS000756 Narcolepsy	https://www.pgscatalog.org/score/PGS000756/
	PGS001150 Sleep duration	https://www.pgscatalog.org/score/PGS001150/
	PGS001978 Sleep duration	https://www.pgscatalog.org/score/PGS001978/
	PGS002196 Sleep duration	https://www.pgscatalog.org/score/PGS002196/
	PGS003480 Sleep duration	https://www.pgscatalog.org/score/PGS003480/
	PGS003764 Sleep duration	https://www.pgscatalog.org/score/PGS003764/
	PGS002144 Trouble falling asleep	https://www.pgscatalog.org/score/PGS002144/
	PGS000908 Insomnia	https://www.pgscatalog.org/score/PGS000908/
	PGS001932 Insomnia	https://www.pgscatalog.org/score/PGS001932/
	PGS002149 Insomnia	https://www.pgscatalog.org/score/PGS002149/
	PGS003319 Insomnia	https://www.pgscatalog.org/score/PGS003319/
	PGS003320 Insomnia	https://www.pgscatalog.org/score/PGS003320/
	PGS003321 Insomnia	https://www.pgscatalog.org/score/PGS003321/
	PGS003322 Insomnia	https://www.pgscatalog.org/score/PGS003322/
	PGS003323 Insomnia	https://www.pgscatalog.org/score/PGS003323/
	PGS003324 Insomnia	https://www.pgscatalog.org/score/PGS003324/
	PGS003325 Insomnia	https://www.pgscatalog.org/score/PGS003325/
	PGS003326 Insomnia	https://www.pgscatalog.org/score/PGS003326/
	PGS003327 Insomnia	https://www.pgscatalog.org/score/PGS003327/
	PGS003328 Insomnia	https://www.pgscatalog.org/score/PGS003328/
	PGS003473 Insomnia	https://www.pgscatalog.org/score/PGS003473/
	PGS003859 Insomnia	https://www.pgscatalog.org/score/PGS003859/
	PGS003204 Sleep apnea	https://www.pgscatalog.org/score/PGS003204/
	PGS003205 Sleep apnea	https://www.pgscatalog.org/score/PGS003205/
	PGS003206 Sleep apnea	https://www.pgscatalog.org/score/PGS003206/
	PGS003207 Sleep apnea	https://www.pgscatalog.org/score/PGS003207/
	PGS003208 Sleep apnea	https://www.pgscatalog.org/score/PGS003208/
	PGS003209 Sleep apnea	https://www.pgscatalog.org/score/PGS003209/
	PGS003210 Sleep apnea	https://www.pgscatalog.org/score/PGS003210/
	PGS003211 Sleep apnea	https://www.pgscatalog.org/score/PGS003211/
	PGS003212 Sleep apnea	https://www.pgscatalog.org/score/PGS003212/
	PGS003213 Sleep apnea	https://www.pgscatalog.org/score/PGS003213/
	PGS003214 Sleep apnea	https://www.pgscatalog.org/score/PGS003214/
	PGS003215 Sleep apnea	https://www.pgscatalog.org/score/PGS003215/
	PGS003216 Sleep apnea	https://www.pgscatalog.org/score/PGS003216/
	PGS003217 Sleep apnea	https://www.pgscatalog.org/score/PGS003217/
	PGS003218 Sleep apnea	https://www.pgscatalog.org/score/PGS003218/
	PGS003479 Sleep apnea	https://www.pgscatalog.org/score/PGS003479/
	PGS003857 Sleep apnea	https://www.pgscatalog.org/score/PGS003857/
	PGS003858 Sleep apnea (BMI adjusted)	https://www.pgscatalog.org/score/PGS003858/
Immune-inflammatory markers	PGS000314 CRP	https://www.pgscatalog.org/score/PGS000314/
	PGS000675 CRP	https://www.pgscatalog.org/score/PGS000675/
	PGS001946 CRP	https://www.pgscatalog.org/score/PGS001946/

	PGS002164 CRP	https://www.pgscatalog.org/score/PGS002164/
	PGS002860 CRP	https://www.pgscatalog.org/score/PGS002860/
	PGS002861 CRP	https://www.pgscatalog.org/score/PGS002861/
	PGS002862-CRP	https://www.pgscatalog.org/score/PGS002862/
	PGS002863 CRP	https://www.pgscatalog.org/score/PGS002863/
	PGS002864 CRP	https://www.pgscatalog.org/score/PGS002864/
	PGS002865 CRP	https://www.pgscatalog.org/score/PGS002865/
	PGS002866 CRP	https://www.pgscatalog.org/score/PGS002866/
	PGS002867 CRP	https://www.pgscatalog.org/score/PGS002867/
	PGS002868 CRP	https://www.pgscatalog.org/score/PGS002868/
	PGS002869 CRP	https://www.pgscatalog.org/score/PGS002869/
	PGS002870 CRP	https://www.pgscatalog.org/score/PGS002870/
	PGS002871 CRP	https://www.pgscatalog.org/score/PGS002871/
	PGS002872 CRP	https://www.pgscatalog.org/score/PGS002872/
	PGS002873 CRP	https://www.pgscatalog.org/score/PGS002873/
	PGS002874 CRP	https://www.pgscatalog.org/score/PGS002874/
	PGS002875 CRP	https://www.pgscatalog.org/score/PGS002875/
	PGS002876 CRP	https://www.pgscatalog.org/score/PGS002876/
	PGS002877 CRP	https://www.pgscatalog.org/score/PGS002877/
	PGS002878 CRP	https://www.pgscatalog.org/score/PGS002878/
	PGS002879 CRP	https://www.pgscatalog.org/score/PGS002879/
	PGS002880 CRP	https://www.pgscatalog.org/score/PGS002880/
	PGS002881 CRP	https://www.pgscatalog.org/score/PGS002881/
	PGS002882 CRP	https://www.pgscatalog.org/score/PGS002882/
	PGS002883 CRP	https://www.pgscatalog.org/score/PGS002883/
	PGS002884 CRP	https://www.pgscatalog.org/score/PGS002884/
	PGS002885 CRP	https://www.pgscatalog.org/score/PGS002885/
	PGS002886 CRP	https://www.pgscatalog.org/score/PGS002886/
	PGS002887 CRP	https://www.pgscatalog.org/score/PGS002887/
	PGS002888 CRP	https://www.pgscatalog.org/score/PGS002888/
	PGS003527 CRP	https://www.pgscatalog.org/score/PGS003527/
	PGS004335 CRP	https://www.pgscatalog.org/score/PGS004335/
	PGS000241 Galanin	https://www.pgscatalog.org/score/PGS000241/
	PGS000242 Galectin 3	https://www.pgscatalog.org/score/PGS000242/
	PGS000249 IL18	https://www.pgscatalog.org/score/PGS000249/
	PGS000251 IL27	https://www.pgscatalog.org/score/PGS000251/
	PGS000252 IL6	https://www.pgscatalog.org/score/PGS000252/
	PGS000254 IL8	https://www.pgscatalog.org/score/PGS000254/
	PGS000255 IL16	https://www.pgscatalog.org/score/PGS000255/
	PGS000253 IL6RA	https://www.pgscatalog.org/score/PGS000253/
	PGS000250 IL1r antagonist protein	https://www.pgscatalog.org/score/PGS000250/
	PGS000287 TNF-R1	https://www.pgscatalog.org/score/PGS000287/
	PGS000288 TNF-R2	https://www.pgscatalog.org/score/PGS000288/
	PGS000223 TNF-R5 (CD40)	https://www.pgscatalog.org/score/PGS000223/

	PGS000238 TNF-R6 (FAS)	https://www.pgscatalog.org/score/PGS000238/
	PGS000289 TNF-ligand14	https://www.pgscatalog.org/score/PGS000289/
	PGS000220 C-C motif chemokine 20	https://www.pgscatalog.org/score/PGS000220/
	PGS000221 C-C motif chemokine 3	https://www.pgscatalog.org/score/PGS000221/
	PGS000222 C-C motif chemokine 4	https://www.pgscatalog.org/score/PGS000222/
	PGS000281 Resistin	https://www.pgscatalog.org/score/PGS000281/
	PGS001036 Epstein barr virus nuclear antigen 1 IgG seropositivity	https://www.pgscatalog.org/score/PGS001036/
	PGS001772 ZEBRA antigen for epstein barr virus	https://www.pgscatalog.org/score/PGS001772/
	PGS000105 Neutrophil count	https://www.pgscatalog.org/score/PGS000105/
	PGS000182 Neutrophil count	https://www.pgscatalog.org/score/PGS000182/
	PGS001173 Neutrophil count	https://www.pgscatalog.org/score/PGS001173/
	PGS001969 Neutrophil count	https://www.pgscatalog.org/score/PGS001969/
	PGS002187 Neutrophil count	https://www.pgscatalog.org/score/PGS002187/
	PGS003545 Neutrophil count	https://www.pgscatalog.org/score/PGS003545/
	PGS003938 Neutrophil count	https://www.pgscatalog.org/score/PGS002938/
	PGS004358 Neutrophil count	https://www.pgscatalog.org/score/PGS004358/
	PGS000097 Lymphocyte count	https://www.pgscatalog.org/score/PGS000097/
	PGS000172 Lymphocyte count	https://www.pgscatalog.org/score/PGS000172/
	PGS001199 Lymphocyte count	https://www.pgscatalog.org/score/PGS001199/
	PGS001965 Lymphocyte count	https://www.pgscatalog.org/score/PGS001965/
	PGS002183 Lymphocyte count	https://www.pgscatalog.org/score/PGS002183/
	PGS002338 Lymphocyte count	https://www.pgscatalog.org/score/PGS002338/
	PGS002370 Lymphocyte count	https://www.pgscatalog.org/score/PGS002370/
	PGS002410 Lymphocyte count	https://www.pgscatalog.org/score/PGS002410/
	PGS002459 Lymphocyte count	https://www.pgscatalog.org/score/PGS002459/
	PGS002508 Lymphocyte count	https://www.pgscatalog.org/score/PGS002508/
	PGS002557 Lymphocyte count	https://www.pgscatalog.org/score/PGS002557/
	PGS002606 Lymphocyte count	https://www.pgscatalog.org/score/PGS002606/
	PGS002655 Lymphocyte count	https://www.pgscatalog.org/score/PGS002655/
	PGS002704 Lymphocyte count	https://www.pgscatalog.org/score/PGS002704/
	PGS003475 Lymphocyte count	https://www.pgscatalog.org/score/PGS003475/
	PGS003936 Lymphocyte count	https://www.pgscatalog.org/score/PGS003936/
	PGS004356 Lymphocyte count	https://www.pgscatalog.org/score/PGS004356/
	PGS000102 Monocyte count	https://www.pgscatalog.org/score/PGS000102/
	PGS000177 Monocyte count	https://www.pgscatalog.org/score/PGS000177/
	PGS001163 Monocyte count	https://www.pgscatalog.org/score/PGS001163/
	PGS001968 Monocyte count	https://www.pgscatalog.org/score/PGS001968/
	PGS002186 Monocyte count	https://www.pgscatalog.org/score/PGS002186/
	PGS002341 Monocyte count	https://www.pgscatalog.org/score/PGS002341/
	PGS002372 Monocyte count	https://www.pgscatalog.org/score/PGS002372/
	PGS002413 Monocyte count	https://www.pgscatalog.org/score/PGS002413/
	PGS002462 Monocyte count	https://www.pgscatalog.org/score/PGS002462/
	PGS002511 Monocyte count	https://www.pgscatalog.org/score/PGS002511/
	PGS002560 Monocyte count	https://www.pgscatalog.org/score/PGS002560/

	PGS002609 Monocyte count	https://www.pgscatalog.org/score/PGS002609/
	PGS002658 Monocyte count	https://www.pgscatalog.org/score/PGS002658/
	PGS002707 Monocyte count	https://www.pgscatalog.org/score/PGS002707/
	PGS003544 Monocyte count	https://www.pgscatalog.org/score/PGS003544/
	PGS003937 Monocyte count	https://www.pgscatalog.org/score/PGS003937/
	PGS004357 Monocyte count	https://www.pgscatalog.org/score/PGS004357/
	PGS000109 Platelet count	https://www.pgscatalog.org/score/PGS000109/
	PGS000186 Platelet count	https://www.pgscatalog.org/score/PGS000186/
	PGS001238 Platelet count	https://www.pgscatalog.org/score/PGS001238/
	PGS001973 Platelet count	https://www.pgscatalog.org/score/PGS001973/
	PGS002191 Platelet count	https://www.pgscatalog.org/score/PGS002191/
	PGS002343 Platelet count	https://www.pgscatalog.org/score/PGS002343/
	PGS002373 Platelet count	https://www.pgscatalog.org/score/PGS002373/
	PGS002415 Platelet count	https://www.pgscatalog.org/score/PGS002415/
	PGS002464 Platelet count	https://www.pgscatalog.org/score/PGS002464/
	PGS002513 Platelet count	https://www.pgscatalog.org/score/PGS002513/
	PGS002562 Platelet count	https://www.pgscatalog.org/score/PGS002562/
	PGS002611 Platelet count	https://www.pgscatalog.org/score/PGS002611/
	PGS002660 Platelet count	https://www.pgscatalog.org/score/PGS002660/
	PGS002709 Platelet count	https://www.pgscatalog.org/score/PGS002709/
	PGS003546 Platelet count	https://www.pgscatalog.org/score/PGS003546/
	PGS003932 Platelet count	https://www.pgscatalog.org/score/PGS003932/
	PGS004352 Platelet count	https://www.pgscatalog.org/score/PGS004352/
	PGS000104 Mean platelet count	https://www.pgscatalog.org/score/PGS000104/
	PGS000179 Mean platelet volume	https://www.pgscatalog.org/score/PGS000179/
	PGS001200 Mean platelet volume	https://www.pgscatalog.org/score/PGS001200/
	PGS002340 Mean platelet volume	https://www.pgscatalog.org/score/PGS002340/
	PGS002412 Mean platelet volume	https://www.pgscatalog.org/score/PGS002412/
	PGS002461 Mean platelet volume	https://www.pgscatalog.org/score/PGS002461/
	PGS002510 Mean platelet volume	https://www.pgscatalog.org/score/PGS002510/
	PGS002559 Mean platelet volume	https://www.pgscatalog.org/score/PGS002559/
	PGS002608 Mean platelet volume	https://www.pgscatalog.org/score/PGS002608/
	PGS002657 Mean platelet volume	https://www.pgscatalog.org/score/PGS002657/
	PGS002706 Mean platelet volume	https://www.pgscatalog.org/score/PGS002706/
	PGS002312 Autoimmune disease	https://www.pgscatalog.org/score/PGS002312/
	PGS002359 Autoimmune disease	https://www.pgscatalog.org/score/PGS002359/
	PGS002384 Autoimmune disease	https://www.pgscatalog.org/score/PGS002384/
	PGS002433 Autoimmune disease	https://www.pgscatalog.org/score/PGS002433/
	PGS002482 Autoimmune disease	https://www.pgscatalog.org/score/PGS002482/
	PGS002531 Autoimmune disease	https://www.pgscatalog.org/score/PGS002531/

RESULTS

Supplementary Table S2. Ranking of predictors exceeding VIP>1 resulting from the partial least squares regression in the whole sample.

Predictor	VIP	Weight
PGS002390 Chronotype (morning)	3.09447	-0.16709
PGS002439 Chronotype (morning)	2.876576	-0.15532
PGS002609 Monocyte count	2.79705	0.151027
PGS002560 Monocyte count	2.771005	0.14962
PGS001163 Monocyte count	2.651995	0.143194
PGS000145 Depression (ICD-10 defined)	2.61386	-0.14114
PGS001055 Chronotype	2.58463	0.139557
PGS003473 Insomnia	2.334601	0.126057
PGS002413 Monocyte count	2.327114	0.125652
PGS002033 Obesity	2.248825	-0.12143
PGS000242 Galectin 3	2.207769	0.119208
PGS001298 Obesity (time to event)	2.119432	-0.11444
PGS002868 CRP	2.080571	-0.11234
PGS003328 Insomnia	2.071593	-0.11186
PGS003326 Insomnia	2.053913	-0.1109
PGS003325 Insomnia	2.052823	-0.11084
PGS000176 Placenta growth factor	2.050911	0.110739
PGS002462 Monocyte count	2.044186	0.110376
PGS002359 Autoimmune disease	1.963675	-0.10603
PGS003764 Sleep duration	1.951027	0.105346
PGS000138 Lifetime Major Depressive Disorder	1.93323	-0.10439
PGS002815 Age menarche	1.895615	0.102354
PGS002488 Chronotype (morning)	1.83371	-0.09901
PGS002635 Chronotype (morning)	1.826356	-0.09861
PGS003937 Monocyte count	1.822955	0.09843
PGS003333 Major depressive disorder	1.773751	-0.09577
PGS002915 Estradiol	1.706948	-0.09217
PGS003552 Sex hormone binding globulin	1.702335	-0.09192
PGS002887 CRP	1.689375	0.091218
PGS002310 Age menarche	1.682469	0.090845
PGS000139 Lifetime Major Depressive Disorder (with recurrence)	1.656181	-0.08943
PGS003586 Preeclampsia	1.653493	0.08928
PGS001020 Social interaction	1.642481	0.088686

PGS003320 Insomnia	1.631377	0.088086
PGS003321 Insomnia	1.631377	0.088086
PGS001946 CRP	1.610316	-0.08695
PGS000220 C-C motif chemokine 20	1.598444	-0.08631
PGS004240 Sex hormone binding globulin	1.593904	0.086063
PGS002127 Age first birth	1.593182	0.086024
PGS002511 Monocyte count	1.592278	0.085975
PGS000102 Monocyte count	1.576345	0.085115
PGS002866 CRP	1.562319	-0.08436
PGS000908 Insomnia	1.510539	-0.08156
PGS002195 Sex hormone binding globulin	1.506312	-0.08133
PGS001913 Length of menstrual cycle	1.484671	-0.08017
PGS001016 Sensitivity/hurt feelings	1.482442	-0.08004
PGS002676 Age menarche	1.481027	0.079968
PGS002480 Age menarche	1.469905	0.079367
PGS000327 Autism spectrum disorder	1.466968	0.079209
PGS000756 Narcolepsy	1.447501	-0.07816
PGS000222 C-C motif chemokine 4	1.430457	0.077237
PGS000907 Major depressive disorder	1.395061	-0.07533
PGS000251 IL27	1.371013	-0.07403
PGS002136 Recent feelings of foreboding	1.36988	-0.07397
PGS002164 CRP	1.355021	-0.07316
PGS001977 Sex hormone binding globulin	1.344514	-0.0726
PGS000757 Narcolepsy	1.335382	-0.0721
PGS002537 Chronotype (morning)	1.330283	-0.07183
PGS003544 Monocyte count	1.32765	0.071686
PGS001091 Loneliness	1.326915	-0.07165
PGS002372 Monocyte count	1.316655	0.071093
PGS002658 Monocyte count	1.312656	0.070877
PGS003580 Major depressive disorder (lifetime)	1.309573	-0.07071
PGS000253 IL6RA	1.293071	-0.06982
PGS002431 Age menarche	1.291623	0.069741
PGS000177 Monocyte count	1.289519	-0.06963
PGS002186 Monocyte count	1.289097	0.069605
PGS002309 Number of kids	1.286126	-0.06944
PGS002869 CRP	1.282935	-0.06927
PGS001866 Endometriosis	1.262398	0.068163

PGS000143 Seen a General Practitioner for nerves, anxiety, tension or depression	1.242511	-0.06709
PGS002626 Number of kids	1.239293	-0.06692
PGS003581 Major depressive disorder (lifetime)	1.236311	-0.06676
PGS003505 Age first birth	1.231918	0.066517
PGS001825 Obesity	1.219343	-0.06584
PGS003447 Endometriosis	1.213042	0.065498
PGS000694 Sex hormone binding globulin	1.206151	-0.06513
PGS003475 Lymphocyte count	1.201831	0.064893
PGS002875 CRP	1.196677	-0.06461
PGS002876 CRP	1.196677	-0.06461
PGS001968 Monocyte count	1.195827	0.064569
PGS003508 Age menarche	1.187996	0.064146
PGS002586 Chronotype (morning)	1.167796	-0.06306
PGS003578 Major depressive disorder (lifetime)	1.164237	-0.06286
PGS002820 Age at menopause	1.154771	-0.06235
PGS002149 Insomnia	1.147464	-0.06196
PGS000675 CRP	1.146524	-0.06191
PGS003859 Insomnia	1.145081	-0.06183
PGS003527 CRP	1.134519	-0.06126
PGS000314 CRP	1.128781	-0.06095
PGS003753 ADHD	1.128596	-0.06094
PGS002129 Length of menstrual cycle	1.126279	-0.06081
PGS002787 Type 1 bipolar disorder	1.113225	-0.06011
PGS002790 Autism spectrum disorder	1.095894	0.059173
PGS000135 Schizophrenia	1.095552	0.059154
PGS003579 Major depressive disorder (lifetime)	1.093973	-0.05907
PGS002144 Trouble falling asleep	1.093954	0.059068
PGS002877 CRP	1.092742	0.059003
PGS002482 Autoimmune disease	1.088512	0.058774
PGS002479 Number of kids	1.084496	-0.05856
PGS000134 Schizophrenia	1.081116	0.058375
PGS003507 Testosterone in females	1.061936	-0.05734
PGS002675 Number of kids	1.059691	-0.05722
PGS003322 Insomnia	1.04318	0.056326
PGS002079 Irregular cycle	1.026797	0.055442
PGS003583 Major depressive disorder (lifetime)	1.004622	-0.05424

Supplementary Table 3. Ranking of predictors exceeding VIP>1 resulting from the partial least squares regression in the MDD sample.

Predictor	VIP	Weight
PGS001163 Monocyte count	2.620997	0.141935
PGS002560 Monocyte count	2.497397	0.135242
PGS000675 CRP	2.493915	-0.13505
PGS002868 CRP	2.467013	-0.1336
PGS002609 Monocyte count	2.466529	0.13357
PGS000138 Lifetime Major Depressive Disorder	2.348414	-0.12717
PGS001946 CRP	2.320174	-0.12564
PGS000314 CRP	2.314715	-0.12535
PGS002869 CRP	2.248379	-0.12176
PGS002866 CRP	2.246868	-0.12168
PGS000176 Placenta growth factor	2.231108	0.120821
PGS002875 CRP	2.222277	-0.12034
PGS002876 CRP	2.222277	-0.12034
PGS002885 CRP	2.153021	-0.11659
PGS002886 CRP	2.153021	-0.11659
PGS002164 CRP	2.097142	-0.11357
PGS002413 Monocyte count	2.075674	0.112404
PGS002786 Bipolar disorder	2.023508	0.109579
PGS002865 CRP	2.021453	-0.10947
PGS002462 Monocyte count	2.019543	0.109364
PGS003473 Insomnia	2.010985	0.108901
PGS002787 Type 1 bipolar disorder	1.999797	0.108295
PGS002033 Obesity	1.974157	-0.10691
PGS000249 IL18	1.972982	0.106843
PGS002658 Monocyte count	1.971553	0.106766
PGS002129 Length of menstrual cycle	1.961717	-0.10623
PGS002884 CRP	1.920569	-0.10401
PGS002511 Monocyte count	1.9098	0.103421
PGS003527 CRP	1.882186	-0.10193
PGS000102 Monocyte count	1.876512	0.101619
PGS002707 Monocyte count	1.851404	0.100259
PGS003544 Monocyte count	1.808489	0.097935
PGS003328 Insomnia	1.79807	-0.09737
PGS000142 Probable Depression	1.777504	-0.09626

PGS000287 TNF-R1	1.752802	-0.09492
PGS002867 CRP	1.699234	-0.09202
PGS003937 Monocyte count	1.632563	0.088408
PGS002463 Neuroticism	1.627622	0.088141
PGS000288 TNF-R2	1.605314	-0.08693
PGS002873 CRP	1.597122	-0.08649
PGS000139 Lifetime Major Depressive Disorder (with recurrence)	1.583364	-0.08574
PGS001055 Chronotype	1.537624	0.083267
PGS002359 Autoimmune disease	1.525399	-0.08261
PGS003480 Sleep duration	1.516511	-0.08212
PGS001968 Monocyte count	1.488305	0.080596
PGS002864 CRP	1.486846	-0.08052
PGS002653 Hypothyroidism	1.470001	-0.07961
PGS000242 Galectin 3	1.46108	0.079122
PGS002482 Autoimmune disease	1.445564	0.078282
PGS002186 Monocyte count	1.436282	0.077779
PGS000193 Major depressive disorder	1.424893	-0.07716
PGS002341 Monocyte count	1.414841	0.076618
PGS001883 Osteroperosis	1.398699	0.075744
PGS001091 Loneliness	1.37845	-0.07465
PGS001020 Social interaction	1.35085	0.073153
PGS002871 CRP	1.342926	-0.07272
PGS002036 Depression	1.327834	-0.07191
PGS001992 Chronotype	1.323739	-0.07168
PGS000908 Insomnia	1.322492	-0.07162
PGS003327 Insomnia	1.316952	-0.07132
PGS003764 Sleep duration	1.314679	0.071194
PGS001829 Depression	1.309145	-0.07089
PGS003507 Testosterone in females	1.308076	-0.07084
PGS002790 Autism spectrum disorder	1.295733	0.070168
PGS001866 Endometriosis	1.280235	0.069329
PGS004356 Lymphocyte count	1.277842	-0.0692
PGS002870 CRP	1.275452	-0.06907
PGS003326 Insomnia	1.264488	-0.06848
PGS003325 Insomnia	1.249484	-0.06766
PGS001913 Length of menstrual cycle	1.225229	-0.06635
PGS003208 Sleep apnea	1.223295	-0.06625

PGS004335 CRP	1.217135	-0.06591
PGS000134 Schizophrenia	1.212838	0.065679
PGS002414 Neuroticism	1.208928	0.065467
PGS002922 Estradiol	1.205176	-0.06526
PGS002079 Irregular cycle	1.187458	0.064304
PGS000327 Autism spectrum disorder	1.186047	0.064228
PGS002823 Age at menopause	1.154645	-0.06253
PGS001298 Obesity (time to event)	1.153754	-0.06248
PGS003215 Sleep apnea	1.145986	0.062059
PGS003552 Sex hormone binding globulin	1.134505	-0.06144
PGS002815 Age menarche	1.130111	0.061199
PGS000240 Follistatin serum levels	1.127246	-0.06104
PGS002915 Estradiol	1.113592	-0.0603
PGS002195 Sex hormone binding globulin	1.11271	-0.06026
PGS003333 Major depressive disorder	1.111851	-0.06021
PGS000253 IL6RA	1.103011	-0.05973
PGS000135 Schizophrenia	1.096664	0.059388
PGS001978 Sleep duration	1.091072	0.059085
PGS000182 Neutrophil count	1.083143	0.058655
PGS002531 Autoimmune disease	1.079963	-0.05848
PGS002702 Hypothyroidism	1.077433	-0.05835
PGS002816 Age menarche	1.076201	0.05828
PGS002817 Age menarche	1.076201	0.05828
PGS002920 Estradiol	1.071348	0.058017
PGS002919 Estradiol	1.069009	0.05789
PGS000759 Hypothyroidism	1.062353	-0.05753
PGS002183 Lymphocyte count	1.061322	-0.05747
PGS002788 Type 2 bipolar disorder	1.052483	0.056995
PGS000756 Narcolepsy	1.051028	0.056916
PGS000136 Schizophrenia	1.048167	0.056761
PGS002586 Chronotype (morning)	1.041485	-0.0564
PGS002196 Sleep duration	1.037183	0.056167
PGS000289 TNF-ligand14	1.028644	0.055704
PGS002561 Neuroticism	1.025229	0.055519
PGS002874 CRP	1.01969	-0.05522
PGS002310 Age menarche	1.003727	0.054355

Supplementary Table 4. Ranking of predictors exceeding VIP>1 resulting from the partial least squares regression in the BD sample.

Predictor	VIP	Weight
PGS002439 Chronotype (morning)	3.56492	-0.19305
PGS002390 Chronotype (morning)	3.480118	-0.18846
PGS002787 Type 1 bipolar disorder	3.266653	-0.1769
PGS000756 Narcolepsy	3.259824	-0.17653
PGS002786 Bipolar disorder	3.003132	-0.16263
PGS000145 Depression (ICD-10 defined)	2.766093	-0.14979
PGS004240 Sex hormone binding globulin	2.638613	0.142889
PGS002488 Chronotype (morning)	2.598059	-0.14069
PGS000220 C-C motif chemokine 20	2.470876	-0.13381
PGS000757 Narcolepsy	2.384331	-0.12912
PGS002144 Trouble falling asleep	2.253884	0.122055
PGS002874 CRP	2.251627	0.121932
PGS000288 TNF-R2	2.148125	0.116328
PGS000287 TNF-R1	2.123103	0.114972
PGS001055 Chronotype	2.069257	0.112057
PGS002372 Monocyte count	2.056492	0.111365
PGS002823 Age at menopause	2.019515	0.109363
PGS002684 Chronotype (morning)	1.971082	-0.10674
PGS002136 Recent feelings of foreboding	1.970138	-0.10669
PGS000251 IL27	1.954304	-0.10583
PGS002877 CRP	1.932465	0.104649
PGS002635 Chronotype (morning)	1.912121	-0.10355
PGS002872 CRP	1.887105	0.102192
PGS003475 Lymphocyte count	1.863595	0.100919
PGS002479 Number of kids	1.855053	-0.10046
PGS002887 CRP	1.806456	0.097825
PGS004356 Lymphocyte count	1.782442	0.096525
PGS001298 Obesity (time to event)	1.776867	-0.09622
PGS002920 Estradiol	1.752148	-0.09488
PGS002127 Age first birth	1.738841	0.094164
PGS002318 Chronotype (morning)	1.678663	-0.09091
PGS003322 Insomnia	1.619539	0.087703
PGS002885 CRP	1.610224	0.087199
PGS002886 CRP	1.610224	0.087199

PGS000143 Seen a General Practitioner for nerves, anxiety, tension or depression	1.595402	-0.0864
PGS003325 Insomnia	1.57433	-0.08526
PGS002309 Number of kids	1.561353	-0.08455
PGS003326 Insomnia	1.559869	-0.08447
PGS000242 Galectin 3	1.55328	0.084115
PGS003859 Insomnia	1.548283	-0.08384
PGS002609 Monocyte count	1.513139	0.081941
PGS001829 Depression	1.499008	0.081176
PGS002815 Age menarche	1.487338	0.080544
PGS003320 Insomnia	1.455682	0.07883
PGS003321 Insomnia	1.455682	0.07883
PGS002560 Monocyte count	1.451564	0.078607
PGS002433 Autoimmune disease	1.414773	-0.07661
PGS003586 Preeclampsia	1.397329	0.07567
PGS002513 Platelet count	1.39285	-0.07543
PGS002704 Lymphocyte count	1.377079	0.074573
PGS003764 Sleep duration	1.341299	0.072635
PGS003333 Major depressive disorder	1.329066	-0.07197
PGS001992 Chronotype	1.321625	0.07157
PGS002759 Depression	1.318096	-0.07138
PGS002310 Age menarche	1.316995	0.071319
PGS002463 Neuroticism	1.310753	-0.07098
PGS002870 CRP	1.302335	0.070525
PGS002922 Estradiol	1.295267	0.070143
PGS000141 Seen a psychiatrist for nerves, anxiety, tension or depression	1.287097	0.0697
PGS002884 CRP	1.275182	0.069055
PGS002915 Estradiol	1.261596	-0.06832
PGS002036 Depression	1.249476	0.067663
PGS002431 Age menarche	1.241234	0.067217
PGS002626 Number of kids	1.237147	-0.067
PGS000177 Monocyte count	1.227324	-0.06646
PGS002506 Hypothyroidism	1.220832	0.066112
PGS003212 Sleep apnea	1.212776	-0.06568
PGS003552 Sex hormone binding globulin	1.2081	-0.06542
PGS000675 CRP	1.199144	0.064937
PGS002113 Depression by trauma	1.198805	-0.06492
PGS002537 Chronotype (morning)	1.196534	-0.0648

PGS002413 Monocyte count	1.196235	0.06478
PGS002359 Autoimmune disease	1.193338	-0.06462
PGS001016 Sensitivity/hurt feelings	1.189754	-0.06443
PGS002336 Hypothyroidism	1.185541	0.064201
PGS002480 Age menarche	1.182511	0.064037
PGS003473 Insomnia	1.18242	0.064032
PGS003506 Length of menstrual cycle	1.180679	0.063937
PGS000694 Sex hormone binding globulin	1.175885	-0.06368
PGS002676 Age menarche	1.148452	0.062192
PGS003319 Insomnia	1.131468	0.061272
PGS002653 Hypothyroidism	1.128601	0.061117
PGS002806 Gestational duration	1.123938	0.060865
PGS001977 Sex hormone binding globulin	1.112216	-0.06023
PGS002820 Age at menopause	1.11082	-0.06015
PGS003328 Insomnia	1.104371	-0.05981
PGS000314 CRP	1.085005	0.058756
PGS001883 Osteoporosis	1.078966	-0.05843
PGS002128 Length of menstrual cycle	1.077372	0.058343
PGS003447 Endometriosis	1.058998	0.057348
PGS002655 Lymphocyte count	1.037433	0.05618
PGS002033 Obesity	1.036918	-0.05615
PGS000217 Adrenomedullin	1.035824	-0.05609
PGS002149 Insomnia	1.032331	-0.0559
PGS001825 Obesity	1.030043	-0.05578
PGS000222 C-C motif chemokine 4	1.021844	0.055336
PGS002675 Number of kids	1.016545	-0.05505
PGS002183 Lymphocyte count	1.012127	0.05481
